# The role of PET/CT in detection of gastric cancer recurrence

**DOI:** 10.1186/1471-2407-9-73

**Published:** 2009-03-01

**Authors:** Sung Hoon Sim, Yu Jung Kim, Do-Youn Oh, Se-Hoon Lee, Dong-Wan Kim, Won Jun Kang, Seock-Ah Im, Tae-You Kim, Woo Ho Kim, Dae Seog Heo, Yung-Jue Bang

**Affiliations:** 1 Department of Internal Medicine, Seoul National University Hospital, Seoul National University College of Medicine, Seoul, Korea; 2 Department of Nuclear medicine, Seoul National University Hospital, Seoul National University College of Medicine, Seoul, Korea; 3 Department of Pathology, Seoul National University Hospital, Seoul National University College of Medicine, Seoul, Korea; 4 Cancer Research Institute, Seoul National University College of Medicine, Seoul, Korea

## Abstract

**Background:**

In the course of surveillance of gastric cancer recurrence after curative resection, contrast CT scan is used in general. However, new findings from CT scan are not always confirmatory for the recurrence. In this case, we usually use short-term follow up strategy or therapeutic intervention with clinical decision. Recently, the use of fusion Positron Emission Tomography/Computed Tomography (PET/CT) is increasing. The purpose of this study is to evaluate the efficacy and usefulness of PET/CT for detecting recurrence of gastric cancer after curative resection.

**Methods:**

Fifty two patients who received curative resection of gastric cancer and had undergone PET/CT and contrast CT for surveillance of recurrence until Dec 2006 in Seoul National University Hospital were analyzed retrospectively. Recurrence of gastric cancer was validated by histologic confirmation (n = 17) or serial contrast CT follow up with at least 5 month interval (n = 35). McNemar's test and Fisher's exact test were used to evaluate sensitivity and specificity of PET/CT and contrast CT.

**Results:**

Of 52 patients, 38 patients were confirmed as recurrence. The sensitivity was 68.4% (26/38) for PET/CT and 89.4% (34/38) for contrast CT (p = 0.057). The specificity was 71.4% (10/14) and 64.2% (9/14), respectively (p = 1.0). In terms of the recurred sites, the sensitivity and specificity of PET/CT were similar to those of contrast CT in all sites except peritoneum. Contrast CT was more sensitive than PET/CT (p = 0.039) for detecting peritoneal seeding. Additional PET/CT on contrast CT showed no further increase of positive predictive value regardless of sites. Among 13 patients whose image findings between two methods were discordant and tissue confirmation was difficult, the treatment decision was made in 7 patients based on PET/CT, showing the final diagnostic accuracy of 42.8% (3/7).

**Conclusion:**

PET/CT was as sensitive and specific as contrast CT in detection of recurred gastric cancer except peritoneal seeding. However, additional PET/CT on contrast CT did not increase diagnostic accuracy in detection of recurred gastric cancer. Further studies are warranted to validate the role of PET/CT in detection of gastric cancer recurrence.

## Background

Gastric cancer is the fourth most common cancer worldwide with approximately 930,000 new cases and 700,000 deaths per year [1]. The incidence of stomach cancer is high in Asia, and it is the most common cancer in Korea [2].

Early gastric cancer can be cured by complete resection, while advanced disease often recurs in 40~60% of patients after surgery [3,4]. To detect cancer recurrence, various methods such as tumor markers, endoscopy or imaging studies have been used. However, tumor markers cannot localize the recurrence site and endoscopy cannot detect extra-luminal recurrence [5].

At present, the most frequently used method for the detection of gastric cancer recurrence is contrast-enhanced computed tomography (contrast CT) [5,6]. However, CT has a notable limitation on diagnosis of malignancy because it uses the size criteria. Therefore, it cannot reflect the presence and viability of tumor precisely.

Fluorine 18 (18F) fluorodeoxyglucose (FDG) positron emission tomography (PET), in contrast with CT, is a tool that reflects cancer metabolism and cell biology. Recently, FDG-PET combined with CT (Fusion PET/CT) has been introduced and is expected to give us more precise anatomical data with metabolic information. Fusion PET/CT has been reported to be useful for staging of lung, colorectal, breast, lymphoma, head and neck cancer and for detecting recurrence of lung, colorectal, thyroid, and breast cancer [7-14]. However, in contrast to the other cancer, it has been reported that FDG uptake of gastric cancer cell is relatively poor and the PET image has a limitation on the detection of recurred gastric cancer [15,16].

The definitive method for diagnosis of gastric cancer recurrence is the pathologic confirmation. However, getting adequate tissues is often difficult because either recurred tumor size is very small or it is deeply located or too close to great vessels or organs for needle biopsy.

In clinical practice, it is hard to make treatment decision when gastric cancer recurrence is suspicious in contrast CT but tissue confirmation is difficult. In this case, additional PET/CT could give us more information on the detection of recurrence. Therefore, we conducted this study to evaluate efficacy and usefulness of PET/CT for the detection and confirmation of recurred gastric cancer after curative resection.

## Methods

All information was collected and analyzed retrospectively. We screened all the patients with gastric cancer who received curative resection and had subsequently undergone contrast CT and PET/CT for the surveillance of recurrence between Apr. 2004 and Dec. 2006 in Seoul National University Hospital. The patients were enrolled when the cancer recurrence can be validated by tissue confirmation or by the change of lesions on contrast CT follow up of at least 5-month interval. Basically, all the patients had undergone routine follow up with 3 to 6 month interval after curative resection. Regardless of PET/CT, the diagnostic contrast CT scan was performed with 120 kVP, 120 mA, 5 mm thickness and 90 ml contrast media, which were adjusted to body weight.

### PET/CT Imaging

All scans were performed by PET/CT system (Philips Gemini, DA best, Netherlands). The patients were asked to fast for at least 4 hours before undergoing PET/CT and 555–740 MBq (15–20 mCi; 0.22 mCi/kg body weight) of FDG was administered intravenously 1 hour prior to imaging. CT was performed prior to PET, and the resulting data were used to generate an attenuation correction map for PET. Five-millimeter-thick sections were obtained at 50 mA (but adjusted for body thickness) and 120 kVp from the skull base to the mid-thigh. Next, PET was performed with a 5-min emission acquisition per imaging level and the images were reconstructed.

### Data analysis and Statistical methods

Fusion PET/CT was reviewed by a nuclear medicine physician who was aware of patients' information on clinical findings and other imaging results. All lesions in PET/CT and contrast CT were categorized as suspicious, negative or equivocal lesions. Exo and Endo luminal recurrence was categorized as locoregional recurrence and the other patents of recurrence categorized as metastasis. In statistical analysis, the equivocal lesions were considered as negative lesions. Recurrence of gastric cancer was validated by histologic confirmation or serial contrast CT follow up. It was regarded as negative lesion when there was no change between CT images of at least the 5 month interval. Overall and site-specific sensitivity, specificity, positive and negative predictive values were calculated. McNemar's test and Fisher's exact test were used to evaluate the efficacy of PET/CT and contrast CT.

### Ethics

The study protocol was reviewed and approved by the institutional review board of Seoul National University Hospital. The recommendations of the Declaration of Helsinki for biomedical research involving human subjects were also followed.

## Results

### Patients

52 patients in total were enrolled. Characteristics of the patients are presented in Table 1. Forty three patients were male. In terms of initial pathologic staging of initial gastric cancer, 11.5% of the patients were in Ia, 21.1% in Ib, 19.2% in II, 25.0% in IIIa, 7.7% in IIIb and 15.4% in IV. According to TNM stage, all stage IV patients revealed TxN3M0 status. Adenocarcinoma was the predominant histologic type of all resected specimens (90.4%, n = 47). Among these patients, signet ring cell type was in 7.7% (n = 4) patients.

**Table 1:** Patient Characteristics

Characteristics		No. (%)
Age	Median	62
	Range	33~80
Sex	Male	43 (82.6)
	Female	9 (17.3)
Pathologic stage	Ia	6 (11.5)
	Ib	11 (21.1)
	II	10 (19.2)
	IIIa	13 (25.0)
	IIIb	4 (7.7)
	IV	8 (15.4)
Pathology	Adenocarcinoma	47 (90.4)
	signet ring cell type	4 (7.7)
	Unknown	1 (1.9)
Adjuvant chemotherapy	Yes	35 (67.3)
	No	17 (32.6)
Operation	Total gastrectomy	26 (50)
	Subtotal gastrectomy	26 (50)
Interval between CT and PET/CT	Median	9 days
	Range	0~45 days

### Detection of Recurrence by fusion PET/CT and contrast CT

The interval between contrast CT and fusion PET/CT ranged from 0 days to 45 days, with a median interval of 9 days. Among 52 patients, recurrence was confirmed in 38 patients by pathologic diagnosis or contrast CT follow up of at least 5-month intervals (17 by pathologic confirmation, 35 by contrast CT follow up, Table 2). In 38 recurred cases, most of the recurrence sites were lymph nodes(n = 20) and peritoneum (n = 15). Seven patients had recurrence in remnant stomach or anastomosis sites and six in the liver. Each one patient showed lung, subcutaneous and pleural metastasis. The sensitivity and specificity according to recurrence site are described in Table 3. The sensitivity and specificity between two methods were not statistically significant except in detection of peritoneal carcinomatosis. The contrast CT was more sensitive than fusion PET/CT (p = 0.039) in detecting peritoneal carcinomatosis.

**Table 2:** Recurrence and its specific site

Variables		No.
Recurrence	Yes	38
	No	14
Pathological confirmation		17
Clinical confirmation by image		35
Recurrence site	Locoregional recurrence	**7**
	(Remnant stomach or anastomosis site)	
	Distant metastasis	37
	Lymph-node	20
	Liver	6
	Other site (bone, skin, etc.)	3
	Peritoneum	15

### Fusion PET/CT in addition to contrast CT

The overall positive predictive value of PET/CT on contrast CT was 89.7%. The site specific positive predictive values are presented in Table 3 and Table 4. The additional fusion PET/CT did not increase the positive predictive value statistically.

**Table 3:** Overall and site specific sensitivity and specificity of contrast CT and fusion PET/CT

Site		Contrast CT	Fusion PET/CT	p-value
Overall	Sensitivity (%)	89.4(34/38)	68.4(26/38)	0.057*
	Specificity (%)	64.2(9/14)	71.4(10/14)	1.0*
	PPV(%)	87.1(34/39)	86.6(26/30)	1.0
Remnant stomach or anastomosis site	Sensitivity (%)	42.85(4/7)	100(7/7)	0.13*
	Specificity (%)	95.5(43/45)	93.3(42/45)	1.0*
	PPV(%)	60.0(3/5)	70.0(7/10)	1.0
Lymph-node	Sensitivity (%)	90(18/20)	70(14/20)	0.21*
	Specificity (%)	87.5(28/32)	96.8(31/32)	0.25*
	PPV(%)	81.8(18/22)	93.3(14/15)	0.63
Peritoneum	Sensitivity (%)	86.6(13/15)	46.6(8/15)	0.039*
	Specificity (%)	91.9(34/37)	94.2(35/37)	1.0*
	PPV(%)	82.3(13/16)	80.0(8/10)	1.0
Liver	Sensitivity (%)	50.0(3/6)	66.6(4/6)	1.0*
	Specificity (%)	100(46/46)	97.8(45/46)	1.0*
	PPV(%)	100(3/3)	80.0(4/5)	1.0

*: calculated by McNemar's test

**Table 4:** Positive predictive value of contrast CT alone and of combination of the two methods

Recurrence site	PPV(%) in Contrast CT +*	PPV(%) in Fusion PETCT +* and contrast CT +*	P-value
Overall recurrence	89.7(35/39)	88.0(22/25)	1.00
Lymph-node	81.8(18/22)	92.8(13/14)	0.62
Peritoneum	72.2(13/18)	85.7(6/7)	0.63
Remnant stomach or anastomosis	60.0(3/5)	75.5(3/4)	1.00
Liver	80.0(4/5)	100(3/3)	0.37

*: There is a lesion which is suspicious for recurrence

**Figure 1:**
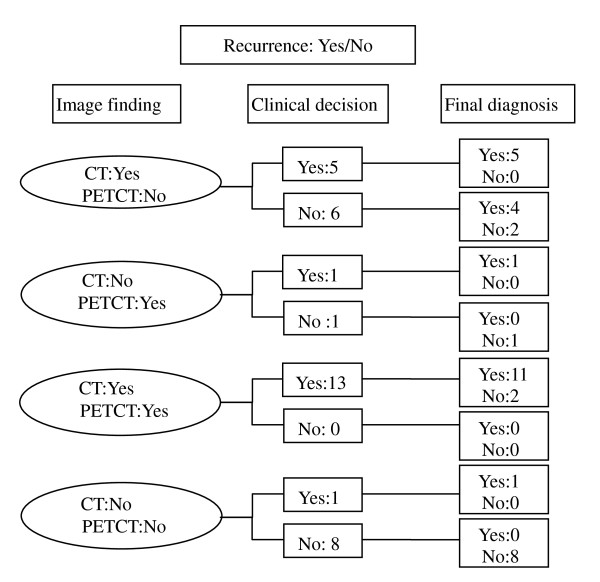
**Treatment decision by findings from fusion PET/CT when tissue confirmation is impossible**. When the image findings between the two methods were discordant, treatment decision was made according to the PET/CT findings in 7 out of 13 cases (Figure 1). But its final accuracy was 42.8% (3/7).

**Figure 2:**
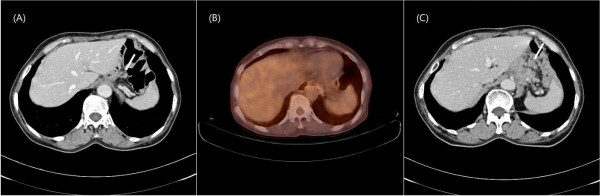
**Contrast CT and PET/CT with lymph node recurrence**. (A) Prominent lymph node in contrast CT suggesting recurrence (arrow) (B) PET/CT finding without hypermetabolic lesions suggesting absence of tumor recurrence (C) Increased previous lesion (arrow) after 6 months.

When the tissue confirmation is impossible, clinical decision and its final diagnosis by contrast CT image follow-up is presented in figure 1. When the image findings between the two methods were discordant, treatment decision was made according to the PET/CT findings in 7 out of 13 cases (Figure 1 and Figure 2). But its final accuracy was 42.8% (3/7). Age and stage distribution in these patients are presented in Table 5. Older and higher stage patients had a tendency of increased recurrence of gastric cancer regardless of results of the fusion PET/CT findings.

**Table 5:** Age and stage distribution in patients with discordant findings but no tissue confirmation

	Recurrence		
Stage	No	Yes	Ratio(Yes/No)
Ix	4	7	1.8(7/4)
II	2	6	3(6/2)
IIIx	1	11	11(11/1)
IV	2	2	1(2/2)
Total	9	26	2.9(26/9)

Age(mean ± SD)	57.0 ± 15.4	62.8 ± 11.1	

Abbreviation: SD, standard deviation

## Discussion

Our study showed that PET/CT was as sensitive and specific as contrast CT in the detection of gastric cancer recurrence except in peritoneal carcinomatosis. Despite this result, additional PET/CT on contrast CT did not give us more accurate information to confirm cancer recurrence.

There are some reports about the role of FDG-PET or fusion PET/CT in the evaluation of gastric cancer recurrence after curative resection, but the results are inconsistent. Jadvar, et al. reports that FDG PET might be useful in the post-therapy evaluation of recurrent disease[17]. Park, et al. also suggested that PET/CT might have a role for detecting recurrence in post-operative patients with gastric cancer[18]. On the other hand, De Potter, et al. reported that FDG-PET might not be suitable as a primary tool for follow up due to its moderate accuracy[19].

In the aspect of sensitivity, fusion PET/CT alone was as sensitive as contrast CT in most recurred sites according to our study. However, the statistical significance is marginal (p = 0.057) and the sensitivity of PET/CT showed inferior tendency compared to contrast CT. Moreover, PET/CT was inferior to contrast CT for detection of peritoneal recurrence, which corresponds with other previous reports [10,20].

These might be attributed to the method of CT validation that could make the ability of contrast CT overestimated. The other explanation would be due to the low metabolic activity of recurred gastric cancer. The interpretation of PET/CT is on both metabolic status of lesion and its anatomical location on non-contrast CT. It is well known FDG avidity depends on histologic type [15]. Signet ring cell type or mucinous type is known to have low FDG avidity. However, only a small portion of signet ring cell cancer patients was included in the study population. And the FDG avidity has not been known yet in recurred gastric cancer.

In the aspect of the confirmation of recurrence, the benefits of additional PET/CT on contast CT were low. This may be because either additional PET/CT has little benefit on prediction of gastric cancer recurrence or the study population is too small to reveal the substantial benefits. However, considering its high cost and small benefits, the usefulness of additional PET/CT on contrast CT is unsatisfactory.

Some reports showed that clinical information including age, tumor size, histologic type, the number of involved LN could be the risk factors for gastric cancer recurrence[21,22]. Our study also showed similar results of increasing tendency of recurrence in higher stages regardless of results of fusion PET/CT (Table 5). Therefore, clinical factors such pathological stage could be more helpful to making treatment decision than PET/CT findings.

Our study has a few limitations. First, not all the recurred cases were confirmed by pathologic diagnosis. the validation of recurrence by contrast CT may have caused physicians to overestimate the detection rate of contrast CT. In addition, 5-month interval may not be enough time to confirm the absence of recurrence. Second, it is a retrospective study with small study population. In our data, although about a half of total patients was early stage patients, the recurrence rate was extremely high. However, although the possibility of selection bas, recurrence to non-recurrence ratio showed the increasing trend of recurrence rate as the stage get advanced in the patients with discordant findings (Table 5).

Despite these deficiencies, our study has significance in giving us evidence of the role of fusion PET/CT in post operative surveillance and in the clinical decision-making process. Further prospective studies enrolling large populations are needed to establish the role of fusion PET/CT in detection of gastric cancer recurrence.

## Conclusion

In conclusion, the role of fusion PET/CT in detection and confirmation of recurrence of gastric cancer is limited. Further well-designed prospective study is needed to establish the role of fusion PET/CT in detection of gastric cancer recurrence

## Competing interests

The authors declare that they have no competing interests.

## Authors' contributions

SHS designed the concept of this study, performed the statistical analysis and drafted the manuscript. YJK S-HL D-WK S-AI collected the data. D-YO participated in its design and coordination, critically revised the manuscript. WJK collected the data and critically revised the manuscript. T-YK WHK DSH Y-JB critically revised the manuscript. All authors read and approved the final manuscript.

## Pre-publication history

The pre-publication history for this paper can be accessed here:

http://www.biomedcentral.com/1471-2407/9/73/prepub
